# Integrate and boost: bioscaffolds nurture the cardiac regenerative paradigm

**DOI:** 10.1186/s13287-015-0184-0

**Published:** 2015-09-23

**Authors:** Jozef Bartunek, Marc Vanderheyden, Atta Behfar

**Affiliations:** Cardiovascular Center, OLV Hospital, Moorselbaan 164, 9300 Aalst, Belgium; Division of Cardiovascular Diseases, Center for Regenerative Medicine, Mayo Clinic, 200 1st St SW, Rochester, MN 55902 USA

## Abstract

The traditional cardiac regenerative paradigm using non-modified adult stem cells with various routes of delivery into the myocardial target has thus far yielded unconvincing clinical outcomes. Besides factors related to heterogeneity in trial methodology, inter-patient variability and the rare incidence of adult stem cells with intrinsic repair potency underscore the importance of further optimization and standardization of regenerative platforms. Cardiac tissue engineering seizing upon the advances of cellular, molecular, and biomaterial development is shaping the next generation of the regenerative paradigm and thereby fostering disruptive curative treatments in heart failure.

## Introduction

In a recent article in *Stem Cell Research & Therapy*, Gálvez-Montón et al. [1] provide new evidence that neurovascular integration of acellular pericardial-derived scaffold may induce cardiac repair. Ischemia-induced myocyte loss is associated with degradation of the extracellular matrix and induction of replacement fibrosis as the primary trigger of adverse ventricular remodeling and heart failure progression. Cell-based regenerative interventions are under investigation as an adjunct to current standards of care in two main settings. If employed acutely after myocardial infarction, cell-based therapy is thought to facilitate myocardial recovery through a cardioprotective pathway. Conversely, in the setting of established cardiac dysfunction, the restorative impact of cell-based therapy aborts pathological remodeling and contributes to the restoration of healthy tissue. Although various clinical trials have demonstrated the safety and feasibility of these approaches, varied signals of benefits between trials have prompted careful evaluation and optimization of existing strategies.

Cardiac tissue engineering and biomaterial scaffolds offer an alternative strategy to regenerate damaged myocardium. They are applied as a patch or as an injection to overcome the hostile ischemic milieu either to facilitate the transplanted cell survival or to promote endogenous repair by facilitating electromechanical coupling and neovasculogenesis [2, 3]. This is key in ensuring the transport of nutrients and functional integration of the scaffold. Using a cell-free pericardial scaffold, the present study provides biological proof of concept for such an approach in a large animal model of acute myocardial infarction [1]. It demonstrates neovascularization and nerve formation in a cell-free pericardial bioscaffold preparation implanted early after experimental myocardial infarction. Detailed immunohistochemistry and electron microscopy analysis demonstrate the functional neovascularization without adjunctive growth factor stimulation. Neovascularization with intraluminal erythrocytes has been observed across the entire thickness of the scaffold away from the host myocardium. Furthermore, authors report de novo nerve sprouting [1]. At the ultrastructural level, these neuronal cells contain structural organelles consistent with the differentiated neuronal afferent cells. The underlying mechanism and signaling were not elucidated here. However, in contrast to synthetic scaffolds, the porous structure of prepared pericardium features preserved biological tunnels, which in the setting of hypoxia likely provide the necessary cues for the host progenitor cells facilitating their migration and scaffold integration [4]. Because the scaffold delivery here was performed early after the myocardial infarction where abundant inflammatory and reparative signals exist, it remains unclear whether a similar degree of neurovascular integration would occur in chronic myocardial infarction. In addition, the lack of a control group prevents the broader translation of infarct size reduction and effects on overall cardiac structure and function.

Aside from the inherent limitations of this biological proof-of-concept experiment, there are several implications for the framework of cardiac regeneration. The traditional cardiac regenerative paradigm using non-modified adult stem cells with various routes of delivery into the myocardial target has thus far yielded unconvincing clinical outcomes [5]. Factors related to heterogeneity in trial methodology, inter-patient variability, and the rare incidence of adult stem cells with intrinsic repair potency underscore the importance of further optimization and standardization of regenerative platforms [5]. Optimization efforts span all levels of the regenerative paradigm (Table 1). Moving beyond heterogenous biologics such as bone marrow mononuclear cells, ongoing clinical trials have employed cell-sorting strategies to boost the endogenous regenerative potential. This next-generation strategy either purifies unique populations of autologous or allogeneic progenitors [6] or achieves anatomical matching with propagation of resident cardiac stem cells [7]. Furthermore, identification of signals that govern cardiogenic specification facilitated the development of cardiopoiesis technology used to boost the regenerative impact of patient-derived stem cells [8].

**Table 1:** Optimization efforts for the cardiac regenerative paradigm

Cell populations	Optimization strategy	Expected impact	Progress to date
Purified cells, mesenchymal stem cells (MSCs), and endothelial progenitor cells (EPCs)	Well-defined population	Repair effect of well-defined progenitors with potential allogeneic off-shelf therapy	• Synergistic use of MSCs and cardiac progenitor cells (CPCs) is currently under investigation
			• Strong signals of benefit with EPCs
CPCs	Anatomical matching	Boosting intrinsic regenerative pool	• Two phase II trials completed
			• Further studies with allogenic populations are under way
Functional lineage matching	Growth factor-guided lineage specification	Boosting paracrine effects together with intrinsic regenerative pool	• Early signals of efficacy
			• This paradigm is under phase III investigation
Delivery techniques	Needle design	Increased retention and cell survival	Curved needle design shown to improve the cell retention
	Procedural guidance		
Myocardial niche modification	Enhanced homing signaling	Boosting cell retention and endogenous repair	Paracrine influence of progenitors achieves enhanced stem cell content in the perivascular stem cell niche within the heart
Tissue engineering	Bioscaffolds	Facilitate cell survival and endogenous repair	Significant advances in tissue processing and three-dimensional printing have paved the way for cell-free approaches for cardiac regeneration

Use of acellular approaches to achieve cardiac regeneration has evolved out of stem cell science but aims to achieve regeneration without the cell-to-cell variability and high manufacturing costs associated with cell therapy. Here, delivery of restorative exogenous signals through the use of scaffolds, proteins, or microRNA aims to boost intrinsic repair function of the heart by modulating the interplay between reactive inflammatory response and stem cell recruitment into the ischemic tissue. In this regard, manipulation of the myocardial microenvironmental appears to boost the stem cell recruitment into the perivascular niche and enhance the efficacy of autologous stem cell delivery [9].

Beyond cell-based or cell-free strategies that boost the endogenous myocardial response, procedural optimization to augment the retention of biologics relies on the development of novel delivery devices or methods to optimize procedural guidance [10]. Alternatively, engineered biomaterial scaffolds may help to overcome challenges currently attributed to cell retention or host tissue niche modification [2]. The present study provides a biological proof of concept that pericardial scaffolds can integrate with host tissue in the setting of acute myocardial infarction, creating neurovascular networks in the epicardial space. Although implementation of this particular approach early after infarction is clinically impractical, integration in chronic models of heart failure may provide a novel approach to regeneration. The observations in this study pave the road for further studies to explore the functional effects of bioscaffold alone or in combination with various cell types.

## Conclusion

Cardiac tissue engineering seizing upon the advances of cellular, molecular, and biomaterial development is shaping the next generation of the regenerative paradigm and thereby fostering disruptive curative treatments in heart failure.
